# X-ray patterns of COVID-19 in patients presenting to Lady Reading Hospital, Peshawar, Pakistan

**DOI:** 10.12669/pjms.37.1.3435

**Published:** 2021

**Authors:** Tahira Nishtar, Nosheen Noor, Shandana Latif Khan

**Affiliations:** 1 Tahira Nishtar, FCPS. Department of Radiology, Lady Reading Hospital-Medical Teaching Institute, Peshawar, Pakistan; 2 Nosheen Noor, FCPS. Department of Radiology, Lady Reading Hospital-Medical Teaching Institute, Peshawar, Pakistan; 3 Shandana Latif Khan, MBBS. Resident, Department of Radiology, Lady Reading Hospital-Medical Teaching Institute, Peshawar, Pakistan

**Keywords:** COVID-19, Portable X-ray, Chest X-ray findings

## Abstract

**Objective:**

To determine the pattern of COVID-19 on chest radiograph in patients presenting to Lady Reading Hospital, Peshawar, Pakistan.

**Methods:**

This prospective observational study was conducted on 178 consecutive swab positive COVID-19 patients presenting to Lady Reading Hospital, Peshawar, Pakistan from 15^th^ March to 15^th^ June 2020. Patients of all ages and both genders were included. Chest X-rays performed by portable radiography unit were viewed for different patterns by two consultant radiologists independently and results were analyzed using IBM SPSS 20.

**Results:**

Out of 178 patients 134 were male. Mean age was 55.67 years. Radiographic patterns observed were ground glass haze without or with reticulation and/or consolidation (45.5 % and 33.2% respectively) and predominant consolidation either alone or in combination with ground glass haze or other findings (27.1% collectively). Peripheral distribution pattern was seen in 69.1% of patients with bilateral findings in 84.3%. Further categorization was based on pulmonary zonal demarcation with changes most commonly involving four zones (33.1%) i.e., the lower and mid zones bilaterally.

**Conclusion:**

Portable chest radiography is an essential supporting tool for assessing different patterns in COVID-19 infection. The most common pattern observed is alveolar opacities with predominant peripheral distribution either unilateral or more frequently bilateral, starting from the lower and mid zones extending to the upper zones and becoming diffuse with disease progression.

## INTRODUCTION

COVID-19, caused by SARS-COV-2, declared a pandemic by world health organization (WHO) in March 2020, has had a global impact since it first emerged in WUHAN, CHINA in late December 2019.^1^ In Pakistan the first case was reported on 26^th^ February 2020 in Karachi.^2^ As of July 2, 2020, about 10,533,779 confirmed cases of COVID-19 have been reported worldwide, resulting in 512,842 deaths in 216 countries.^3^ In Pakistan, 217,809 cases of COVID-19 have been confirmed and 4,473 deaths till date with increasing daily numbers of people testing positive for Corona virus.^4^

Chest radiograph though less sensitive than CT chest, is the first line imaging modality used for COVID-19 patients, with portable chest radiography units used for ease of decontamination and eliminating the need of patient transfer.^5^-^9^ Most of radiological literature focuses on chest CT role and manifestations in COVID -19. According to some studies performed in China, CT has achieved a diagnostic sensitivity even higher than initial RT-PCR swab test^10^ but due to infection control issues, decontamination and lack of CT availability in certain parts of the world, portable chest radiography is the most frequently used imaging modality for identification and follow up of lung manifestations of COVID-19. The American College of Radiology notes that CT decontamination required after scanning COVID-19 patients could disrupt radiological services and portable radiography may be considered to reduce risk of cross contamination.^11^

The rationale of the study was to highlight the different radiographic patterns of COVID-19 which is an essential parameter in both disease diagnosis and prognosis.

## METHODS

This prospective observational study were conducted on an initial series of consecutive 178 patients presenting with COVID-19 infection to Lady Reading Hospital (LRH), Peshawar, Pakistan from 15^th^ March to 15^th^ June 2020. The research protocol was approved by institutions ethics committee on 18^th^ June 20202 under reference number484/LRH. Patients of all ages and both genders admitted in isolation ward, high dependency units (HDU) and intensive care units (ICU) were included in the study. Portable chest radiography was performed and viewed for radiographic patterns. A proforma was devised including patient’s demographics, presenting symptoms, co-morbidities and X- ray findings. Each chest X- ray was viewed by two consultant radiologists independently. Results were analyzed using IBM SPSS 20 version.

## RESULTS

Total number of patients included in the study are 178. Male to female ratio was almost 3:1 with mean age of 55.6 years. Commonest clinical presentation noted was fever, cough and shortness of breath in 88.2% of patients (Table-I). In 57.9% of patients there is no associated co morbidity. In the remaining, diabetes mellitus and hypertension were the commonest comorbidities (25.9 and 20.3% respectively) (Table-II).

**Table-I:** Patient demographics & clinical features.

Variable	N=178	Percentage (%)
***Age (years):***		
Range	12-90	
Mean	55.67	
***Gender:***		
Males	134	75.3
Females	44	24.7
***Presenting symptoms:***		
Fever, cough & SOB	158	88.8
Cough & SOB	12	6.7
Cough	2	1.1
SOB	2	1.1
Cough & fever	1	0.6
Cough, SOB & diarrhea	1	0.6
Diarrhea	1	0.6
Fever, cough, SOB & irritability	1	0.6

SOB = shortness of breath.

**Table-II:** Comorbidities.

Comorbidity	Frequency	Percentage
None	103	57.9
DM	20	11.2
DM & HTN	14	7.9
HTN	7	3.9
Hepatitis C	5	2.8
HTN & IHD	4	2.2
Obesity	3	1.7
Asthma	2	1.1
COPD	2	1.1
DM & hepatitis C	2	1.1
DM, HTN & asthma	2	1.1
DM, HTN & CKD	2	1.1
DM & obesity	2	1.1
HTN & stroke	2	1.1
DM & asthma	1	0.6
DM, HTN & epilepsy	1	0.6
DM, HTN, hepatitis C & anemia	1	0.6
HTN & hepatitis C	1	0.6
Ischemic heart disease	1	0.6
IHD, DM & HTN	1	0.6
IHD, HTN & obesity	1	0.6
Hepatitis B	1	0.6

Total	178	100.0

DM = diabetes mellitus, HTN= Hypertension, IHD = ischemic heart disease, COPD = chronic obstructive pulmonary disease, CKD = chronic kidney disease.

X-Ray patterns seen frequently were ground glass haze without or with reticulation and/or consolidation (45.5 % and 33.2% respectively) and predominant consolidation either alone or in combination with ground glass haze and reticulation or other rare findings (27.1% collectively). (Table-III)

**Table-III:** X-Ray findings.

Variable	N=178	Percentage (%)
Normal X-Ray	5	2.8
***Pattern:***		
GGH	81	45.5
GGH & reticulation	39	21.9
Consolidation	19	10.7
GGH & consolidation	18	10.1
GGH, consolidation & reticulation	7	3.9
Reticulation	3	1.7
Consolidation & soft nodules	1	0.6
Consolidation, nodules & reticulation	1	0.6
Consolidation & reticulation	1	0.6
GGH, consolidation & pleural effusion	1	0.6
GGH, nodules & reticulation	1	0.6
Soft nodules	1	0.6
***Distribution:***		
Peripheral	123	69.1
Diffuse	49	27.5
Central	1	0.6
***Laterality:***		
Bilateral	150	84.3
Right side	13	7.3
Left side	10	5.6
***Zone:***		
Middle & lower	105	59
Upper, middle & lower	59	33.1
Lower	7	3.9
Middle	2	1.1
***Number of zones/scores:***		
Four zones	59	33.1
Three zones	32	18.0
Five zones	30	16.9
Six zones	26	14.6
Two zones	24	13.5
One zone	2	1.1

GGH = ground glass haze.

Peripheral distribution was seen in 69.1% of patients with diffuse pattern in 27.5%. Bilateral changes seen in 84.3% of patients, unilateral changes in 12.9 % with right sided predominance. Middle and lower zone involvement was commonest i.e. 59%, all three zones (upper, middle and lower) were involved in 33.1%, isolated lower and middle zone involvement was seen in 3.9% and 1.1% of patients respectively. As far as number of zones is concerned four zone involvement was seen in 33.1%, three zones in 18%, five zones in 16.9%, six zones in 14.6%, two zones in 13.5% and one zone in 1.1% of patients. Normal chest radiograph noted in 2.8% patients. (Table-III)

## DISCUSSION

Imaging findings in COVID-19 are most commonly of atypical organizing pneumonia, often with bilateral, peripheral and basal predominant distribution.^7^ Different patterns observed in the study were ground glass haze in the early phase with increasing density as the disease progresses. Next stage is the development of heterogeneous dense lobar consolidation becoming homogenous and multi lobar as the disease progresses.

For ease of assessment of degree of lung involvement by disease process, its progression and resolution, a scoring system was devised based on zonal demarcation of lungs, with three zones on each side, i.e. the upper, the middle and the lower zones, each occupying about one third height of the lungs. Arbitrary demarcations of the zones are: from apices to the anterior border of second rib is the upper zone, the middle zone is up to the anterior border of the fourth rib and below that is the lower zone up to the level of diaphragms. The lung zones do not correspond to the lobes for example the lower zone on right includes middle and lower lobes.^12^ Hence, solitary zone involvement given a score of one with maximum score going up to 6.

Most of the patients presenting to LRH were critically ill being admitted to the high dependency and intensive care units and mobile chest radiography was used for imaging. The national guidelines of radiological society of Pakistan (RSP) has also recommended the use of chest radiography for severe and critically ill patients.^13^ It is pertinent to note that majority of patients presenting to emergency department of LRH had a score of at least four, on the base line X-ray. Lady Reading Hospital being the largest tertiary care hospital in Khyber Pukhtunkhwa province of Pakistan, offers free of cost imaging services in both emergency department, in-patient and bed side portable X-ray services. All imaging and reports are available on picture archiving and communication system (PACS).

In the COVID 19 pandemic mobile X-ray unit is the preferred imaging modality used worldwide due to ease of decontamination and most suitable for critically ill patients on support.^5^-^7^ Serial X-rays were performed on patients who had longer duration of hospital stay. X-ray findings were evaluated in terms of the pattern, distribution, unilateral or bilateral disease and the number of zones involved.

X-ray findings may be normal in early disease and tend to appear about 10-12 days after onset of symptoms and normal CXR were seen in 31% of patients in study performed by Wong et al.^1^,^7^ In our study, only 2.8% patients had normal X-ray at presentation. However, majority of the patients presented with advance disease and an abnormal X-ray on admission.The common findings in COVID-19 on CT include ground glass opacities with or without reticulation and consolidations mostly bilateral, peripheral and involving lower lung fields.^5^

Peripheral distribution is a common feature of COVID 19, on CT chest reported to be up to 86% by Ng et al.^14^ These peripheral opacities can be appreciated on CXR as well.^1^ Diffuse opacities can be seen in advanced disease.^6^ In our study peripheral distribution was the commonest in 69.1%, with diffuse changes seen in 27.5% of patients. The commonest finding in our study was peripheral alveoar opacities, either in the form of ground glass haze or consolidation. Ground glass opacities according to the same author were seen in 86% and consolidation in 62% cases,^15^ whereas, in our study ground glass haze either in isolation or in combination with other findings was seen in 78.7% of patients and consolidation in 27.1% of patients. (Table-III).Rare radiological findings of COVID-19 according to different studies include pleural effusion, cavitation, pneumothorax, solid nodules, pericardial effusion and mediastinal or hilar lymphadenopathy.^5^ Rare findings in our study were pulmonary nodules and pleural effusion. (Table-III)

**Fig.1:**
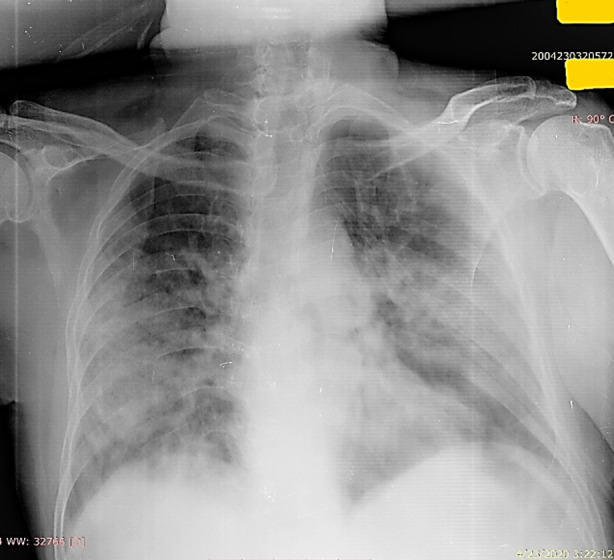
Predominant peripheral heterogeneous opacities involving bilateral middle and lower zones.

**Fig.2:**
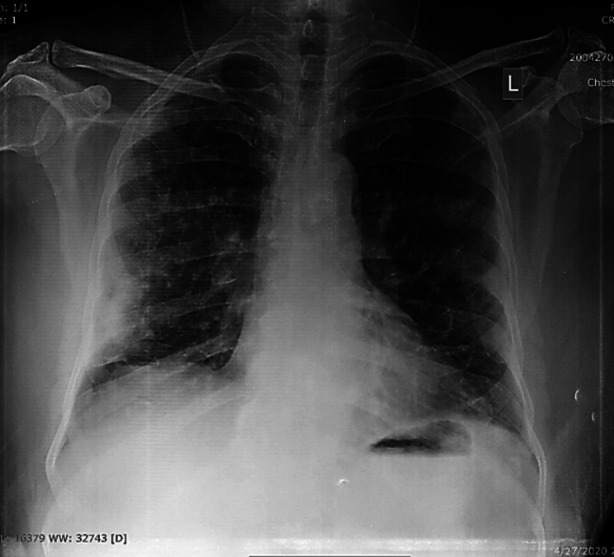
Right middle and lower zone peripheral opacity.

In COVID-19 pneumonia findings are usually bilateral 67%,^6^ however unilateral involvement is seen in equal number of patients according to some studies.^1^ In our study bilateral changes were noted in 84.3% of patients, with unilateral disease (12.9%) presenting as right sided predominance (right side 7.3% and left side 5.6%) (Fig.1 & 2).

Mid and lower zones involvement is a common feature of the disease with upper zone involvement seen rarely.^16^ In our study mid and lower zones were involved in 59%, upper zones extension of disease process seen in severe cases. Isolated upper zone involvement was not seen in any patient. Multiple zone involvement was common, with four zones predominant involvement (33.1%) i.e., lower and middle zones bilaterally, with the left upper zone usually the last one to be involved (Table-III).

Note was made of an interesting phenomenon in few patients with serial X-rays where reversal of pattern was noted with resolution of consolidation from upper to the lower zones and from center to periphery, changing from dense consolidation to GGH with prominent bronchovascular marking and resolution of the opacities (Fig.3a & b).

**Fig.3(a):**
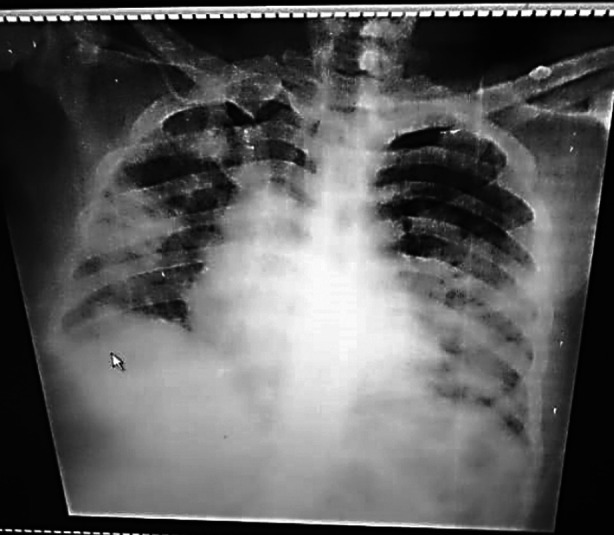
49 years old male patient chest X-ray on presentation showed right three zone and left two zone heterogeneous predominant peripheral opacities.

**Fig.3(b):**
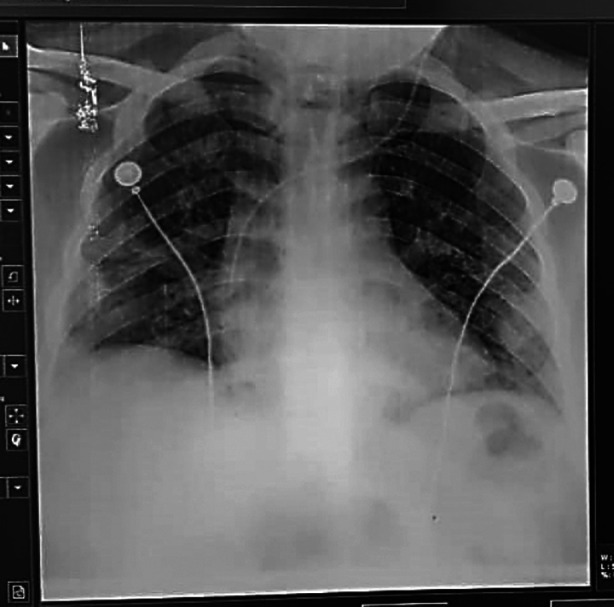
Same patient as in Fig 3(a) chest X-ray at discharge showed significant resolution of the opacities.

Mean age in our study was 55.6 years, compared to 56 years in study performed by Wong et al and 47 years by Guan et al.^16^,^17^ In our study the number of males affected was thrice as females, however, according to a study performed in China more women were affected than men.^17^ The difference in gender prevalence in our study is likely due to cultural factor, majority of Pakistani women being confined indoors and with full outer adornment including face shielding while in public, which plays a protective role in disease transmission.^18^

Common symptoms in study done in China were cough (67.8%) and fever (43.8%) with diarrhea uncommon (3.8%).^17^ Our study also showed the commonest symptoms to be fever, cough and shortness of breath more than 90%, while the uncommon symptoms were diarrhea (Table-I)

According to Wong et al the frequent co-morbidities were diabetes and hypertension, 13 and 20% respectively.^16^ In our study diabetes mellitus and hypertension were the commonest co-morbidities (Table-II). An important finding to note in the study is that 103 out of 178 patients (57%) at presentation did not have an associated co morbidity. In studies performed worldwide, most of the severely ill patients had one or more comorbidity.^19^

A study carried out in China showed that men had more severe disease with worse outcome compared to women, 70.3% mortality in men and 29.7% in women.^20^ In our study the mortality for women was slightly higher than in men, i.e. 68.2% for women and 61.2% for men.

## CONCLUSION

Imaging is essential for assessing severity and disease progression in COVID-19 pneumonia. Radiologists and clinicians should be well versed with imaging manifestations of this infection. Chest X-ray is the first line imaging investigation with portable radiography being the most suitable imaging tool for critically ill patients in this highly contagious viral pandemic. The most important radiographic findings are peripheral alveolar opacities starting from the lower and mid zones extending to the upper zones and becoming diffuse with disease progression.

### Authors Contribution:

**TN** conceived, did manuscript writing, data collection, editing, review, final approval of manuscript and is responsible for integrity of the study.

**NN** did data collection, manuscript writing, statistical analysis and responsible and accountable of study.

**SLK** did data collection and compilation.
